# A Diet With Docosahexaenoic and Arachidonic Acids as the Sole Source of Polyunsaturated Fatty Acids Is Sufficient to Support Visual, Cognitive, Motor, and Social Development in Mice

**DOI:** 10.3389/fnins.2019.00072

**Published:** 2019-02-25

**Authors:** Sarah J. Carlson, Alison A. O’Loughlin, Lorenzo Anez-Bustillos, Meredith A. Baker, Nicholas A. Andrews, Georgia Gunner, Duy T. Dao, Amy Pan, Prathima Nandivada, Melissa Chang, Eileen Cowan, Paul D. Mitchell, Kathleen M. Gura, Michela Fagiolini, Mark Puder

**Affiliations:** ^1^Vascular Biology Program and Department of Surgery, Boston Children’s Hospital, Boston, MA, United States; ^2^Department of Neurology, F.M. Kirby Neurobiology Center, Boston Children’s Hospital – Program in Neuroscience, Harvard Medical School, Boston, MA, United States; ^3^Institutional Centers for Clinical and Translational Research, Boston Children’s Hospital, Boston, MA, United States; ^4^Division of Gastroenterology, Hepatology and Nutrition, Boston Children’s Hospital, Boston, MA, United States; ^5^Department of Pharmacy, Boston Children’s Hospital, Boston, MA, United States

**Keywords:** polyunsaturated fatty acid (PUFA), omega-3 fatty acids, diet, neurocognition, docosahexaenoic acid (DHA), arachidonic acid (AA)

## Abstract

Polyunsaturated fatty acids serve multiple functions in neurodevelopment and neurocognitive function. Intravenous lipid emulsions are administered to children that are dependent on parenteral nutrition to provide the essential fatty acids needed to sustain growth and development. One of these emulsions, derived from fish-oil, is particularly poor in the traditional essential fatty acids, linoleic and alpha-linolenic acids. However, it does contain adequate amounts of its main derivatives, arachidonic acid (ARA) and docosahexaenoic acid (DHA), respectively. This skewed composition has raised concern about the sole use of fish-oil based lipid emulsions in children and how its administration can be detrimental to their neurodevelopment. Using a custom-made diet that contains ARA and DHA as a sole source of polyunsaturated fatty acids, we bred and fed mice for multiple generations. Compared to adult, chow-fed mice, animals maintained on this special diet showed similar outcomes in a battery of neurocognitive tests performed under controlled conditions. Chow-fed mice did perform better in the rotarod test for ataxia and balance, although both experimental groups showed a conserved motor learning capacity. Conversely, mice fed the custom diet rich in DHA and ARA showed less neophobia than the chow-fed animals. Results from these experiments suggest that providing a diet where ARA and DHA are the sole source of polyunsaturated fatty acids is sufficient to support gross visual, cognitive, motor, and social development in mice.

## Introduction

Essential fatty acids (EFA) are structural components of cell membranes and play key roles in cell signaling, immune function, and brain development during gestation and early postnatal life (Innis, 2007). EFA cannot be synthesized in the body from simple carbon precursors and therefore must be consumed in the diet to sustain cell growth and reproduction (Holman, 1958). Traditionally, alpha-linolenic acid (ALA), an omega-3 fatty acid (O3FA) and linoleic acid (LA), an omega-6 fatty acid (O6FA), have been considered to be the main EFA. However, recent studies have shown that their respective downstream metabolites, the long-chain polyunsaturated fatty acids (LC-PUFA) docosahexaenoic acid (DHA) and arachidonic acid (ARA), are sufficient to sustain growth and reproduction in mammals without the development of essential fatty acid deficiency (EFAD) (Gura et al., 2005; Strijbosch et al., 2008; Le et al., 2009; de Meijer et al., 2010; Anez-Bustillos et al., 2017; Harauma et al., 2017).

For children who are unable to consume EFA by means of enteral intake, parenteral nutrition (PN) is the mainstay of caloric support. Despite the fact that PN is life-saving for individuals with insufficient gastrointestinal length and/or function, morbidity associated with PN is significant. Common PN-associated complications include central venous catheter-related bloodstream infections, biliary stasis, and intestinal failure-associated liver disease (IFALD) (Wales et al., 2005). Since lipid emulsions have been linked to the development of this condition, a parenteral fish oil-based lipid emulsion (FOLE) has emerged as a safe and efficacious alternative for its treatment (Gura et al., 2008; Puder et al., 2009; Lam et al., 2014; Premkumar et al., 2014).

Compared to standard soybean oil-based lipid emulsions, FOLE contains less than 2% as ALA and 5% as LA, yet is rich in the downstream metabolites, DHA (12%) and eicosapentaenoic acid (EPA) (Bang et al., 1976; Chen et al., 1996; Nehra et al., 2014). Given the beneficial effects of ALA and LA in brain development and aging, the skewed composition of FOLE in favor of DHA and ARA has raised some concern about a possible detrimental impact to the neurological development of children receiving it for prolonged periods of time (Bourre, 2004).

Both animal and human studies have shown that administration of O3FA is associated with positive neurocognitive and behavioral outcomes (McCann and Ames, 2005). In addition, animal models have shown the benefit of O3FA supplementation following traumatic and anoxic brain injury, anxiety, and cognition (Mills et al., 2011; Wu et al., 2011; Wang et al., 2013; Gonzales et al., 2015; Pusceddu et al., 2015). Animal models of O3FA deficiency during development have shown impaired neurogenesis, visual acuity, and retinal function (Anderson et al., 2005).

In the current study, we sought to determine the effects of a diet enriched with the O3FA DHA and the O6FA ARA and assess whether or not it is sufficient to allow for normal neurocognitive development in a murine model. Specifically, we raised mice across multiple generations on a diet with its sole source of polyunsaturated fat calories from DHA and ARA and examined multiple neurocognitive outcomes in murine adulthood.

## Materials and Methods

### Murine Model and Diets

**FIGURE 1:**
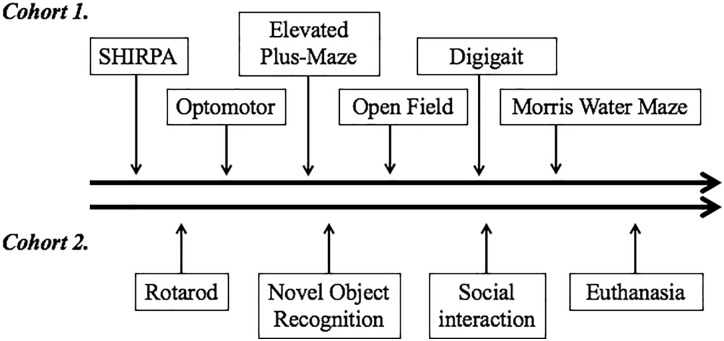
Order of neurobehavioral tests performed. Two different cohorts underwent testing. The timeline on the top depicts tests performed on cohort 1, which started with the least invasive SHIRPA and progressed to the most invasive and anxiety-provoking Morris water maze. The timeline on the bottom depicts the series of tests performed on cohort 2. Following the tests performed, animals from this cohort were euthanized for tissue collection and measurement of fatty acid distribution in different brain regions and cerebellum.

All animal experiments were carried out following approval by the Institutional Animal Care and Use Committee at Boston Children’s Hospital. Adult C57BL/6J mice (Jackson’s Laboratories, Bar Harbor, ME, United States) were maintained in a climate-controlled facility (lights on from 0700 to 1900 h; temperature 22°C ± 1; humidity 45–55%). F0 generation female mice were randomized to one of two dietary groups. One group (DHA/ARA) was fed a diet in which 10% of calories came from fat, with a DHA to ARA ratio of 20:1 and no ALA or LA (7.9% from hydrogenated coconut oil, 2% DHA, and 0.1% ARA) (Dyets #102723, Dyets Inc., Bethlehem, PA, United States). The other group (CHOW) received standard rodent chow (NIH-31 open formula, Charles River Laboratory, Wilmington, MA, United States, or Envigo, Madison, WI, United States) (Table 1). All animals were given *ad libitum* access to food and water. After 4 weeks of dietary treatment, females were housed with an adult male mice to facilitate breeding. Progeny were weaned at age P21 and dietary treatment was continued based on the mother’s assigned group (DHA/ARA vs. CHOW). The first generation progeny (F1) were similarly bred and weaned, and this process was repeated for 10 generations such that colonies of mice were established that had been purely fed DHA/ARA for 10 generations. Mice used for the CHOW group were similarly bred for at least one generation (to avoid unnecessary breeding of a control group for 10 generations as done in the DHA/ARA group). Males from the progeny described above were used at age 3–6 months for a series of neurocognitive tests as detailed below. Handling of mice was kept to a minimum and examiners were blinded to treatment groups. All animals were allowed at least 1 day of rest between experiments. Two separate cohorts were used for the battery of tests (Figure 1).

**Table 1:** Fatty acids and ingredients in experimental diets.

Fatty acid (g/100 g)	CHOW^†^	DHA/ARA^‡^
Linoleic acid (C18:2ω6)	45.8	0
Alpha-linolenic acid (C18:3ω3)	4.27	0
Arachidonic acid (C20:4ω6)	0.26	0.04
Eicosapentaenoic acid (C20:5ω3)	0	0
Docosahexaenoic acid (C22:6ω3)	0.94	0.8
ω-3:ω-6	0.12	20


*^†^The CHOW (NIH-31) diet contained the following: protein 184.2 g/kg, fat (soybean oil) 44.7 g/kg, fiber 40.5 g/kg (Shen et al., 2006). ^‡^The DHA/ARA diet contains the following: casein 140 g/kg, sucrose 100 g/kg, cornstarch 465.7 g/kg, dyetrose 155 g/kg, hydrogenated coconut oil 31.6 g/kg.*

### SHIRPA Test of Morphological and Neurological Function

SHIRPA (SmithKline Beecham, Harwell, Imperial College, Royal London Hospital, phenotype assessment) is a standardized set of experimental procedures used to characterize the phenotype of laboratory mice (Hatcher et al., 2001; Rogers et al., 2001). Animals were observed for an 18-point assessment with regards to a variety of physical characteristics (skin color, coat appearance, whiskers), behaviors (vocalization, biting, lacrimation), and reflexes (corneal, pinnal, righting). Each animal underwent a stage-one SHIRPA assessment as previously described in order to evaluate for differences in overall health and gross neurological behavior between dietary groups (Rogers et al., 2001). Each parameter was scored by an observer that was blinded to the treatment group, based on a pre-determined scale. The sum of parameter scores represented each animal’s total score. Between different mice the viewing chamber was cleaned thoroughly between trials with 10% bleach solution. A total of 20 animals were tested per experimental group.

### Optomotor Test of Visual Acuity

As previously described (Prusky et al., 2004), each mouse was placed on a platform in the center of an arena surrounded by four monitors capable of showing a moving contrast reversal. After 5 min of acclimation, moving black and white vertical stripes of varying width were projected onto the arena walls at a rate of 2 revolutions per minute (rpm). A camera suspended from the ceiling of the chamber captured the animal’s movement, and an examiner, blinded to treatment group, observed whether the mouse’s head showed movement of tracking the visual stimulus. Software (Cerebral Mechanics, New York, NY, United States) was utilized to calculate the threshold of highest visual acuity based on the lowest width of the vertical bars at which the mouse was able to discern movement. Optomotor response (OPT) was defined by the movement of the mouse’s head tracking the moving screen. The OPT of each eye was measured independently as the maximal spatial frequency at which the head was able to track the stimulus (Prusky et al., 2004; Kang et al., 2013). The platform was cleaned thoroughly between trials with 10% bleach solution. For this test, 16 mice were examined in the CHOW group, and 13 in the DHA/ARA group.

### Rotarod Test for Ataxia and Balance

During this test, mice have to keep their balance on a rotating rod. The time (latency) required for each mouse to fall off the rotarod apparatus (Columbus Instruments, Columbus, OH, United States) rotating under continuous acceleration was measured. Mice were given two adaptation trials of 2 min on the apparatus at a constant speed (4 rpm). The following day mice were placed on the apparatus for test trials in which the speed was gradually increased from 4 to 40 rpm, and the time until each mouse fell from the rod was recorded, up to a maximum of 5 min. This was repeated in triplicate for each mouse, with a 5-min rest interval between trials. The apparatus was cleaned thoroughly between trials with 10% bleach solution. Twenty mice were tested per experimental group.

### DigiGait^TM^ Test for Gait Analysis

Gait analysis was performed using the DigiGait^TM^ system (Mouse Specifics Inc., Framingham, MA, United States), which images the underside of the animal as it walks atop a transparent treadmill. Generated digital paw images and dynamic gait signals for each limb describe the posture and kinematics of the animal reflecting strength, balance, and coordination. Each mouse was placed on the treadmill at rest. The treadmill was then started at a speed of 5 cm/s and once the animal was walking, the speed was increased to a final speed of 20 cm/s and the mouse allowed to ambulate for 5 s. DigiGait^TM^ image capturing below the treadmill generated digital images of paw placement, which allowed DigiGait^TM^ Imaging and Analysis Software to calculate a variety of parameters for each animal including the swing, braking, and propulsion of right and left forelimbs and hind limbs. The treadmill was cleaned thoroughly between trials with 10% bleach solution. Fifteen animals were tested per experimental group.

### Open Field Test of Neophobia

Mice that had been handled only minimally prior to experimentation were utilized. Mice were individually placed on the edge of a circular open arena measuring 45 cm in diameter and allowed to roam freely for 20 min. The arena was divided into the outermost periphery (“wall”), the center of the arena (15 cm diameter), and the neutral zone between the center and the wall (25 cm diameter). Video tracking software (Ethovision XT 9.0, Noldus, Netherlands) was used to quantify the distance traveled in each zone, the frequency of zone entries, and the time spent within each zone of the arena by the animal. The arena was housed in a dimly lit room (30 lux) with no noise, and examiners were not present in the room during testing to limit distraction to the mice. The arena was cleaned thoroughly between trials with 10% bleach solution. Twenty mice were tested per experimental group.

### Elevated Plus-Maze Test of Anxiety

Previously unhandled mice underwent this test as previously described (Pellow and File, 1986; Trezza et al., 2009). The apparatus consists of a “+”-shaped maze elevated 90 cm above the floor. Two opposing arms had walls (closed arms; 76 × 6 × 19 cm; L × W × H), and the other two opposing arms had no walls (open arms; 76 × 6 cm). There was a central platform where the four arms crossed (center area; 6 × 6 cm; L × W). Mice were individually placed in the central platform facing toward a closed arm and allowed to explore the maze for 5 min. As subjects freely explored the maze, their activity was recorded by a video camera above the maze. Video tracking software (Ethovision XT 9.0, Noldus, Netherlands) was used to calculate the average velocity and both the frequency of visits and total time spent in the open arms, closed arms, and central platform of the arena. Anxiety-like behavior was measured by the preference for being in open arms, calculated as the percentage of time spent in them (excluding the time spent in the central platform). The apparatus was cleaned thoroughly between trials with 10% bleach solution. Ten mice were tested per experimental group.

### Morris Water Maze Test of Spatial Learning and Memory

The Morris water maze (MWM) tank is a circular 125 cm diameter water-filled pool with walls marked with visual cues above the water line in each of the four quadrants (i.e., N, S, E, W). On day 1 of testing, mice completed a visual platform task in which the water level was 0.5 cm below the level of a 15 cm diameter platform and there was a flag in the platform to aid visibility. Mice were placed in the pool and given up to 90 s to find and climb onto the visible platform. Any mouse not finding the platform in 90 s was guided to the platform by the experimenter and left for 5 s to orient itself. Each mouse received two trials and each trial consisted of starting from each one of the four quadrants in a random order. On day 2, the platform was moved to a different position in the MWM, submerged 1 cm below the water line and the flag removed (hidden platform task). The mice were given up to 90 s to find and climb onto the submerged platform. Any mouse not finding the platform in 90 s was guided to the platform by the experimenter and left for 5 s to orient itself. Each mouse received three trials and each trial consisted of starting from each one of the four quadrants in a random order. On day 3, the mice received two further trials in the hidden platform task. On day 4 (the probe trial), the submerged platform was removed completely from the MWM, and the mouse was allowed to swim freely for 60 s. The time spent in the target quadrant was measured as an additional indicator of spatial learning. On day 5, a reversal task was performed to test flexibility of rule learning. This task was done the same way as on the first day of the hidden trials, with three trials consisting of four quadrant starts on each trial, and a hidden platform placed in a position different from the visible and hidden trials previously undertaken. A camera suspended above the MWM captured each animal’s path within the MWM and computer modeling video tracking software (Ethovision XT 9.0, Noldus, Netherlands) was used to determine the amount of time taken and distance traveled to reach the platform on days 1, 2, 3, and 5. The amount of time and distance traveled in each of the four quadrants was recorded by the software as the animal searched for the platform. For this test, 18 mice were examined in the CHOW group, and 16 in the DHA/ARA group.

### Novel Object Recognition Test

This test consists of two phases (Leger et al., 2013). During the sample (familiarization) phase, the animals were allowed to explore a square arena (37.5 × 37.5 cm) containing two identical objects placed in two of the corners of the arena until they had investigated both objects for a combined total period of 20 s. During the recall (test) phase, animals were given 5 min to explore the same arena containing two objects, one of which was the same used in the sample phase, and one that was a novel object (placed in the same two corners). Ten minutes were allowed to elapse between each phase (intersession interval). The test started with the animal being placed into the center of the arena. Behavior was recorded remotely by a camera and the amount of time in seconds spent interacting with the objects was determined by a blinded investigator. Only time spent with the nose in contact with the object was considered as interaction with that object. Objects in both phases of the test were alternated across trials to control for possible side bias. The objects and arena were cleaned thoroughly between trials with 10% bleach solution. Time spent with the novel object, and its ratio-to-time spent with the familiar (non-novel object) were calculated. Twenty mice were tested per experimental group.

### Three-Chamber Social Interaction Test

Mice were placed in a three-chambered arena with dividing walls made from clear acrylic with rectangular openings that allow access into each chamber. Mice were initially allowed to explore all three chambers for 5 min. After this habituation period, mice were restricted to the center chamber by blocking the access doors to the side chambers. A non-test mouse was placed inside a confined cylinder that allowed for an animal outside to smell the one inside yet preventing them from direct physical contact. This cylindrical container was placed in the corner farthest from the door of either one of the side chambers. In the contralateral side chamber, a similar –but empty– container was placed in the corner farthest from the door. Barriers to the side chambers were then removed and the experimental mouse was allowed to explore freely for 10 min. The time that test mice spent in the side chambers was recorded and used as a marker of sociability. Time spent sniffing the novel mouse and the empty container was used as a more specific marker of social investigation (Yang et al., 2011). Mice were recorded using a video camera and their behavior was tracked using video-tracking software (EthoVision XT 11.5, Noldus, Netherlands). The objects and arena were cleaned thoroughly between trials with 10% bleach solution. For this test, 20 mice were used per experimental group.

### Brain Tissue Harvesting and Fatty Acid Analysis

After completion of the neurobehavioral tests, mice were euthanized under inhaled isoflurane anesthesia. Cerebellum, hippocampus, pons, striatum, and occipital and frontal cortex were harvested, flash frozen, and stored at -80°C for further testing. Total lipids were extracted from the obtained samples following the Folch method (Folch et al., 1957). For this, samples were mixed with heptadecanoic acid (C17:0), which serves as an internal standard (Nu-Chek Prep, Inc., Elysian MN, United States). A biphasic system was then created by adding a mixture of chloroform and methanol (2:1) allowing for lipid separation and extraction. Once separated, the solvent was evaporated under nitrogen conditions to avoid oxidation. Lipids were then saponified with 0.5 N methanolic sodium hydroxide (Ricca Chemical Company, Arlington, TX, United States), and methylated with 12% boron trifluoride (1.5M) in methanol (Acros Organics, Geel, Belgium). Fatty acid methyl ester profiles were then acquired by gas liquid chromatography using an Agilent Technologies 6890N gas chromatograph (Agilent Technologies, Inc., Wilmington, DE, United States) coupled to an Agilent-5975B mass spectrometer equipped with a Supelcowax SP-10 capillary column (Agilent Technologies, Inc.). Results are given as percentage of the total fatty acids. Five mice were used per experimental group.

### Statistical Analysis

Outcome measures are reported as mean ± standard error (SE). Differences between the CHOW and DHA/ARA groups were analyzed according to the complexity of the experiment. Outcomes for the SHIRPA, optomotor, open field, elevated plus-maze, and novel object recognition tests involved comparisons of two independent groups and were compared by Student’s *t*-test. The novel object recognition test was adjusted for the time spent with the non-novel object using analysis of covariance. The remaining outcomes, including the rotarod, DigiGait^TM^, MWM and three-chamber social interaction tests, were analyzed with a repeated-measures general linear model to allow for adjustment of within-mouse correlation among multiple measurements. Rotarod latency times were adjusted for baseline latency. Within-mouse correlation across multiple rotarod and MWM trials was modeled with an autoregressive covariance matrix, reflecting higher correlation for trials closer together and lower correlation for trials farther apart. Correlation among the four limbs of each mouse for the DigiGait^TM^ tests was modeled with a compound symmetric covariance matrix, which assumes equal correlation among the four limbs. The choice of covariance matrix was supported by minimization of Akaike’s Information Criterion (Bozdogan, 1987).

The distribution of each outcome was examined. Those not normally distributed were compared to results after normalizing using the rank-based method of Blom (1958); for all but one outcome, results of this sensitivity analysis were consistent with the original results. The exception was for the elevated plus-maze test of anxiety. Also, in the open field test, an outlier was present in the DHA/ARA group for center-to-wall zone ratio, and in this case, omission of the outlier and comparison by Wilcoxon rank-sum test were consistent with the original *t*-test result. For both of these outcomes, comparisons for the original/complete data are shown as well as the results from the sensitivity analysis. Fatty acid percentages were compared between experimental groups across different brain region by means of a Student’s unpaired *t*-test.

All tests of significance were two-sided with *P* < 0.05 considered statistically significant. Data were analyzed with SAS version 9.4 (SAS Institute, Cary, NC, United States) and plotted with GraphPad Prism 7.0 (GraphPad Software, Inc., San Diego, CA, United States).

## Results

Chow and DHA/ARA adult male mice were evaluated across a variety of behavioral tests that encompassed general health, sensory processing, motor function, anxiety, social interaction, and cognition.

### SHIRPA Test of Morphological and Neurological Function

The SHIRPA protocol was performed to test muscle, cerebellar, sensory, and neuropsychiatric function. No phenotypic or gross behavioral differences were seen between CHOW and DHA/ARA animals. Overall observational scores ranged from 9 to 12 for all animals in both groups, and mean scores were similar between CHOW and DHA/ARA. Similarly, no differences were seen in mean locomotion scores (Table 2).

**Table 2:** Locomotor and overall SHIRPA scores.

Score	Chow	DHA/ARA	*P*-value
Locomotion (mean ± SEM)	15.6 ± 0.9	15.2 ± 1.0	0.77
Overall (mean ± SEM)	10.7 ± 0.3	10.4 ± 0.2	0.37


*P-values are from Student’s *t*-test.*

### Optomotor Test of Visual Acuity

**FIGURE 2:**
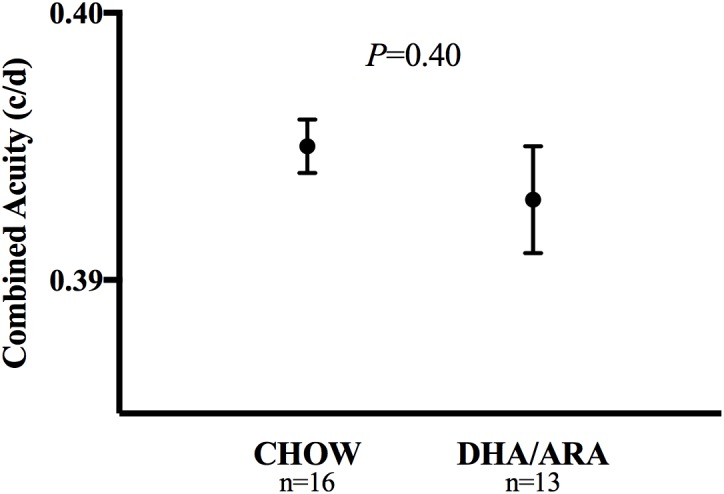
Optomotor test of visual acuity. Shown are mean ± standard error. *P*-values are from Student’s *t*-test.

The OPT was tested as a measure of visual acuity in all animals. Visual acuity was similar between groups, with CHOW and DHA/ARA demonstrating similar mean acuity scores (0.395 ± 0.001 c/d vs. 0.393 ± 0.002 c/d, respectively, *P* = 0.40) (Figure 2).

### Motor Function Tests

**FIGURE 3:**
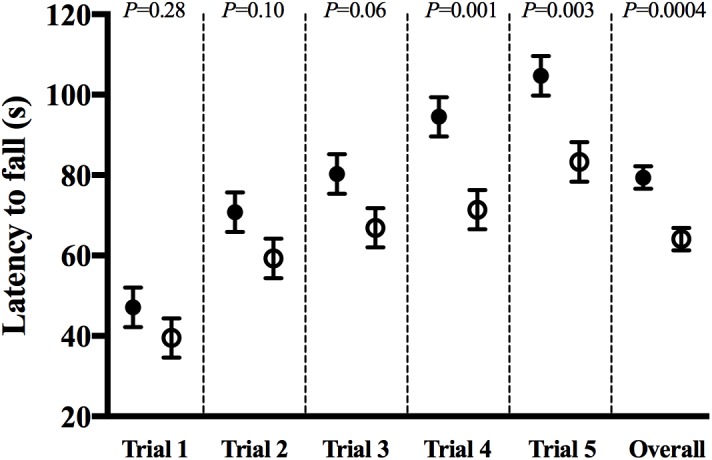
Rotarod test for ataxia and balance: latency to fall in each successive trial (1–5) and overall group effect. CHOW group represented in filled circles, DHA/ARA in empty circles. Differences between CHOW and DHA/ARA groups for each trial were compared by a repeated-measures general linear model, adjusted for baseline (trial 1) latency to fall.

The rotarod test was used to assess motor coordination. Rotarod test scores were different between groups. After adjusting for baseline (trial 1) latency times and the effect of trial number, mice in the CHOW group had significantly higher latency times than those in the DHA/ARA group (*P* = 0.0004). On average, mice receiving DHA/ARA (64.1 ± 2.8) were 15.3 ± 4.1 s faster to fall than mice receiving CHOW (79.4 ± 2.8) (Figure 3).

**FIGURE 4:**
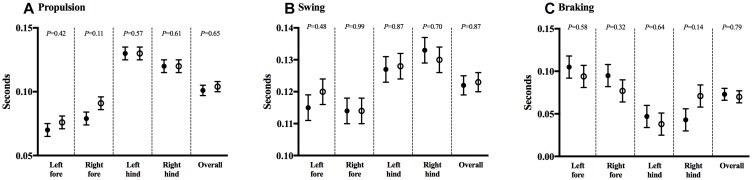
DigiGait^TM^ test for gait analysis. **(A)** Propulsion, **(B)** swing, and **(C)** braking durations. Shown are mean ± standard error. CHOW group represented in filled circles, DHA/ARA in empty circles. Differences between CHOW and DHA/ARA groups for each limb were compared by a repeated-measures general linear model.

Gait analysis allowed for the measurement of several postural and kinematic parameters that reflected the animals’ strength, balance, and coordination. No mean differences were noted in propulsion (0.101 ± 0.004 vs. 0.104 ± 0.004, *P* = 0.65), swing (0.122 ± 0.003 vs. 0.123 ± 0.003, *P* = 0.87), or braking time (0.073 ± 0.007 vs. 0.070 ± 0.007, *P* = 0.79), between CHOW and DHA/ARA, respectively. Limb-specific comparisons were likewise similar and are shown in Figure 4A–C.

### Anxiety Assays

**FIGURE 5:**
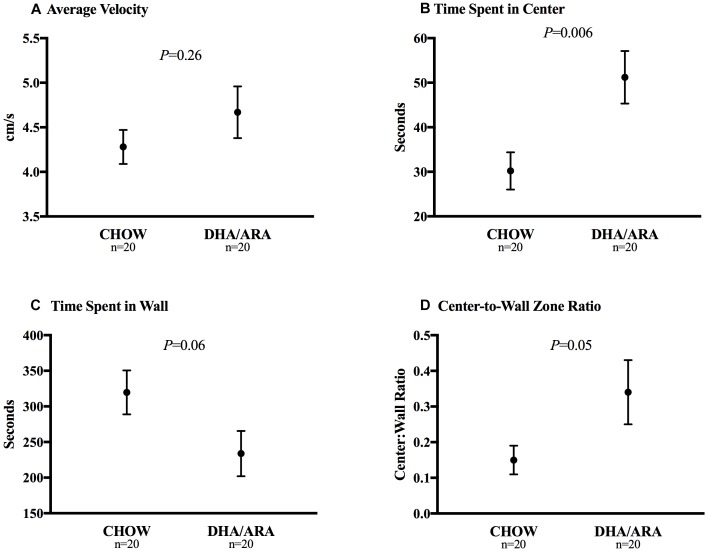
Open field test of neophobia. **(A)** Average velocity. **(B)** Time spent in center zone. **(C)** Time spent in wall zone. **(D)** Center-to-wall zone ratio. Shown are mean ± standard error. *P*-values are from Student’s *t*-test.

The open field test was used to assess neophobia and activity levels (Stanford, 2007). Average velocity was similar between groups (4.28 ± 0.19 cm/s in CHOW vs. 4.67 ± 0.29 cm/s in DHA/ARA, *P* = 0.26) (Figure 5A). On average, DHA/ARA animals spent a greater amount of time in the center zone of the arena when compared with CHOW animals (51.2 ± 5.9 s vs. 30.2 ± 4.2 s, *P* = 0.006) (Figure 5B). CHOW animals tended to spend more time in the wall zone, although the mean difference was not statistically significant (319.7 ± 30.8 s vs. 233.8 ± 31.8 s, *P* = 0.06) (Figure 5C). Overall, the mean center-to-wall zone ratio was higher in DHA/ARA animals (0.34 ± 0.09 vs. 0.15 ± 0.04, *P* = 0.05), although this mean was skewed somewhat by the presence of one high outlier (ratio = 1.9) in the DHA/ARA group (Figure 5D). After omitting the outlier, the mean center-to-wall zone ratio was 0.26 ± 0.03 (*P* = 0.03 for the comparison to CHOW).

**FIGURE 6:**
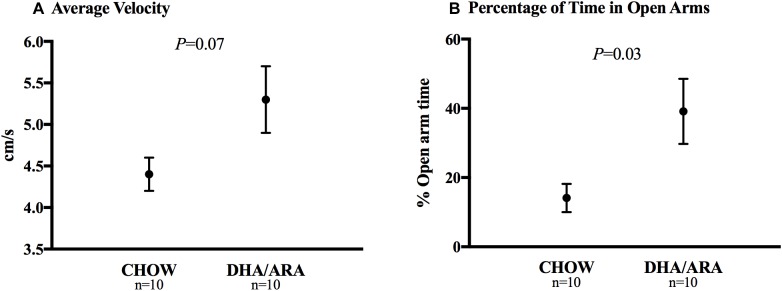
Elevated plus-maze test of anxiety. **(A)** Average velocity and **(B)** percentage of time spent in open arms. Shown are mean ± standard error. *P*-values are from Student’s *t*-test (after normalizing the outcome, *P* = 0.13).

The elevated plus-maze test is used to assess anxiety-related behavior in rodent models of central nervous system disorders. Animals in the DHA/ARA group showed a statistically non-significant higher mean velocity (5.3 ± 0.4 cm/s) compared to CHOW (4.4 ± 0.2 cm/s), *P* = 0.07 (Figure 6A). Compared to CHOW, DHA/ARA animals spent a greater proportion of time on average in the open arms (39.1 ± 9.4% vs. 14.1 ± 4.1%, *P* = 0.03) (Figure 6B). However, this result may be biased due to the skewed distribution of the outcome (*P* = 0.13 after normalizing).

### Learning and Memory Tests

**FIGURE 7:**
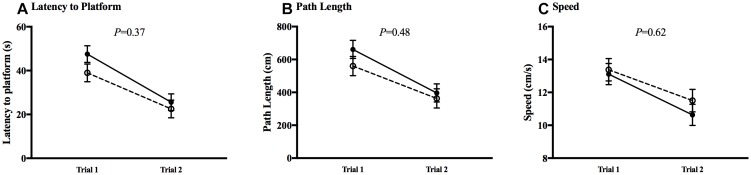
Morris water maze, visible trials. **(A)** Latency to platform. **(B)** Path length. **(C)** Speed. Shown are mean ± standard error for each trial, by group. CHOW group represented in filled circles, DHA/ARA in empty circles. There are no statistical differences between groups for either trial, based on a repeated-measures general linear model.

**FIGURE 8:**
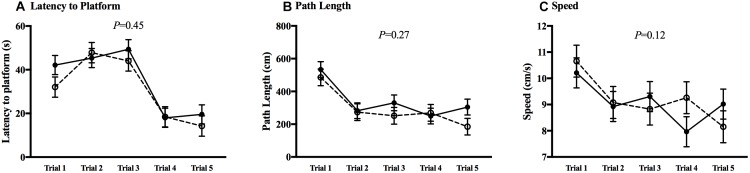
Morris water maze, hidden trials. **(A)** Latency to platform. **(B)** Path length. **(C)** Speed. Shown are mean ± standard error for each trial, by group. CHOW group represented in filled circles, DHA/ARA in empty circles. There are no statistical differences between groups for all trials, based on a repeated-measures general linear model.

The MWM was utilized to test spatial learning and memory. All animals in both CHOW and DHA/ARA groups experienced a decrease from first to last trial (*P* < 0.01), in both visible (Figure 7) and hidden (Figure 8) trials for latency to platform, speed, and path length. There were no statistical differences between groups in any of the five trials for any MWM outcomes.

**FIGURE 9:**
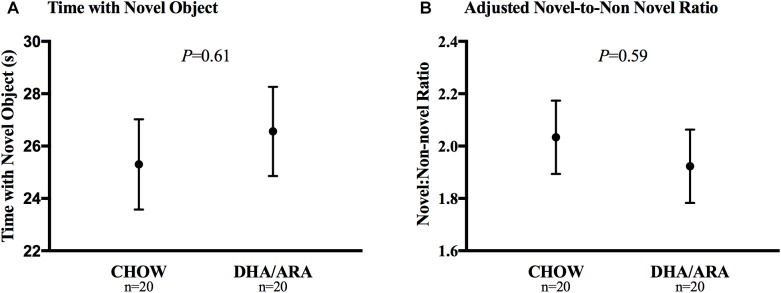
Novel object recognition test. **(A)** Time with novel object. **(B)** Adjusted novel-to-non novel object ratio. Shown are mean ± standard error. *P*-values are from Student’s *t*-test and analysis of covariance.

The novel object recognition test aimed to assess the short-term working memory. Animals in both groups spent a similar amount of time exploring the novel object (25.3 ± 1.7 s vs. 26.6 ± 1.7 s, CHOW and DHA/ARA, respectively), *P* = 0.61 (Figure 9A). After adjusting for time spent with the non-novel object, there were no statistically significant differences between groups (2.03 ± 0.14 vs. 1.92 ± 0.14, CHOW and DHA/ARA, respectively), *P* = 0.59 (Figure 9B).

### Three-Chamber Social Interaction Test

**FIGURE 10:**
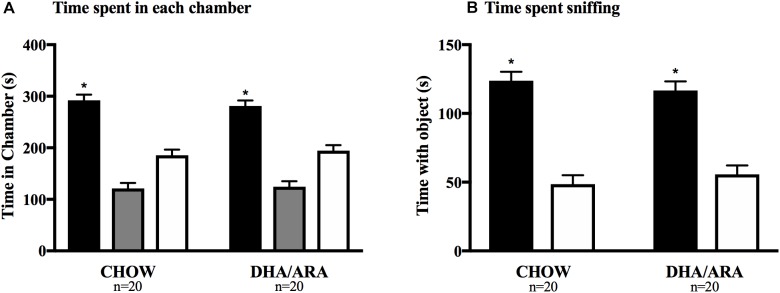
Three-chamber Social Interaction test. **(A)** Time spent in each chamber (black bars represent side chambers with novel mouse; gray bars represent middle chambers; and white bars represent chambers without a mouse). **(B)** Time spent sniffing novel mice (black bars) and empty container (white bars). Shown are mean ± standard error. The asterisks (^∗^) represent *P* < 0.001 for the main effect (CHOW and DHA/ARA combined) comparing time spent in chambers with novel mouse and the middle and empty chambers, from a repeated-measures general linear model.

Social affiliation of mice was tested using the 3-chamber sociability test. Mice in both groups spent a significantly longer amount of time in the chamber with the novel mouse compared to either of the other chambers (*P* < 0.0001), as well as more time sniffing the novel mouse (*P* < 0.0001). There were no differences between the two experimental groups with regards to time spent in the side chamber with the novel mouse (292.3 ± 10.8 s vs. 281.1 ± 10.8 s, CHOW and DHA/ARA, respectively), *P* = 0.46; time spent in the middle chamber (121.1 ± 10.8 s vs. 124.4 ± 10.8 s, CHOW and DHA/ARA, respectively), *P* = 0.83; and time spent in the side chamber without the novel mouse (185.6 ± 10.8 s vs. 194.5 ± 10.8 s, CHOW and DHA/ARA, respectively), *P* = 0.56 (Figure 10A). Similarly, there were no differences between groups in time spent sniffing the novel mouse (123.8 ± 6.5 s vs. 116.6 ± 6.5 s, CHOW and DHA/ARA, respectively), *P* = 0.44; and time spent sniffing the empty container (48.5 ± 6.5 s vs. 55.5 ± 6.5 s, CHOW and DHA/ARA, respectively), *P* = 0.45 (Figure 10B).

### Fatty Acid Distribution

Significant changes in fatty acid composition were seen in the different brain regions (Table 3). Overall, mice fed with the DHA/ARA diet had significantly higher levels of O3FA including EPA and DHA with significantly lower levels of O6FA including LA and ARA.

**Table 3:** Percentage fatty acid distribution of different brain regions and cerebellum.

	Cerebellum		Hippocampus		Pons		Striatum		Occipital		Frontal	
						
	Chow	DHA/ARA	Chow	DHA/ARA	Chow	DHA/ARA	Chow	DHA/ARA	Chow	DHA/ARA	Chow	DHA/ARA
*C14:0*	0.48 ± 0.02	0.46 ± 0.03	0.49 ± 0.01	0.42 ± 0.08	0.46 ± 0.07	0.57 ± 0.05	0.38 ± 0.03	0.35 ± 0.02	0.41 ± 0.03	0.67 ± 0.19	**0.32 ± 0.04**	**0.55 ± 0.05**
*C16:0*	25.2 ± 0.46	26.37 ± 0.32	**26.13 ± 0.2**	**28.33 ± 0.35**	20.57 ± 0.64	21.36 ± 0.29	**27.33 ± 0.37**	**29.02 ± 0.28**	28.46 ± 0.47	28.87 ± 0.84	28 ± 0.34	28.17 ± 0.43
*C16:1*	1.33 ± 0.11	1.68 ± 0.12	1.83 ± 0.09	1.98 ± 0.1	1.36 ± 0.19	1.37 ± 0.11	1.47 ± 0.08	1.55 ± 0.05	1.56 ± 0.05	2.14 ± 0.45	**1.35 ± 0.09**	**1.64 ± 0.05**
*C18:0*	22.47 ± 0.08	22.33 ± 0.07	**24.09 ± 0.16**	**23.15 ± 0.17**	21.87 ± 0.47	20.81 ± 0.24	**23.03 ± 0.06**	**22.68 ± 0.11**	23.29 ± 0.19	23.56 ± 0.66	23.11 ± 0.11	23.22 ± 0.05
*C18:1ω9*	**19.78 ± 0.42**	**21.54 ± 0.34**	16.82 ± 0.44	17.41 ± 0.46	**25.48 ± 0.45**	**29 ± 0.41**	16.51 ± 0.39	15.85 ± 0.41	16.05 ± 0.68	16.14 ± 0.77	**15.33 ± 0.21**	**16.5 ± 0.24**
*C18:1ω7*	4.2 ± 0.05	4.27 ± 0.02	2.71 ± 0.04	2.8 ± 0.06	4.18 ± 0.21	4.27 ± 0.1	3.27 ± 0.08	3.07 ± 0.06	3.07 ± 0.06	2.95 ± 0.1	3.2 ± 0.08	3.29 ± 0.04
*C18:2ω6*	**0.88 ± 0.03**	**0.13 ± 0.01**	**0.7 ± 0.05**	**0.16 ± 0.03**	**0.57 ± 0.02**	**0.1 ± 0.01**	**0.61 ± 0.01**	**0.14 ± 0.01**	**0.6 ± 0.03**	**0.18 ± 0.01**	**0.64 ± 0.03**	**0.16 ± 0.01**
*C19:0*	0.08 ± 0.00	0.06 ± 0.01	0.08 ± 0.00	0.07 ± 0.01	**0.11 ± 0.01**	**0.09 ± 0.00**	0.06 ± 0.00	0.05 ± 0.02	0.08 ± 0.01	0.07 ± 0.01	0.06 ± 0.00	0.08 ± 0.02
*C20:0*	0.28 ± 0.03	0.26 ± 0.02	0.2 ± 0.02	0.16 ± 0.01	0.68 ± 0.03	0.62 ± 0.02	**0.17 ± 0.01**	**0.1 ± 0.01**	0.15 ± 0.02	0.13 ± 0.01	0.13 ± 0.01	0.13 ± 0.01
*C20:1ω9*	1.6 ± 0.17	1.6 ± 0.09	**0.76 ± 0.07**	**0.52 ± 0.06**	3.5 ± 0.21	3.4 ± 0.16	**0.76 ± 0.09**	**0.45 ± 0.06**	0.59 ± 0.11	0.46 ± 0.05	0.54 ± 0.05	0.56 ± 0.05
*C20:1ω7*	0.3 ± 0.03	0.29 ± 0.02	0.17 ± 0.01	0.14 ± 0.01	0.74 ± 0.04	0.68 ± 0.03	0.17 ± 0.02	0.13 ± 0.01	0.13 ± 0.02	0.13 ± 0.01	**0.13 ± 0.01**	**0.16 ± 0.01**
*C20:2ω6*	**0.18 ± 0.01**	**0**	**0.11 ± 0.01**	**0.01 ± 0.01**	**0.23 ± 0.02**	**0.02 ± 0.01**	**0.1 ± 0.01**	**0.01 ± 0.01**	**0.07 ± 0.01**	**0**	**0.11 ± 0.01**	**0**
*C20:3ω9*	**0.05 ± 0.00**	**0.09 ± 0.01**	**0.13 ± 0.01**	**0.46 ± 0.04**	0.13 ± 0.01	0.28 ± 0.07	**0.09 ± 0.01**	**0.3 ± 0.02**	**0.11 ± 0.01**	**0.32 ± 0.05**	**0.08 ± 0.01**	**0.31 ± 0.03**
*C20:3ω6*	**0.62 ± 0.02**	**0.29 ± 0.01**	**0.61 ± 0.03**	**0.36 ± 0.01**	**0.78 ± 0.01**	**0.38 ± 0.02**	**0.6 ± 0.01**	**0.39 ± 0.01**	**0.61 ± 0.02**	**0.42 ± 0.01**	**0.57 ± 0.00**	**0.39 ± 0.01**
*C20:4ω6*	**6.87 ± 0.04**	**3.49 ± 0.08**	**9.54 ± 0.19**	**5.81 ± 0.1**	**6.07 ± 0.13**	**3.21 ± 0.1**	**8.57 ± 0.19**	**5.37 ± 0.12**	**9.39 ± 0.33**	**5.58 ± 0.33**	**8.87 ± 0.19**	**5.13 ± 0.19**
*C20:5ω3*	**0.08 ± 0.01**	**0.44 ± 0.01**	**0.07 ± 0.02**	**0.56 ± 0.08**	**0.03 ± 0.00**	**0.31 ± 0.02**	**0.03 ± 0.00**	**0.38 ± 0.02**	**0.03 ± 0.00**	**0.54 ± 0.05**	**0.04 ± 0.00**	**0.35 ± 0.02**
*C22:0*	0.26 ± 0.02	0.24 ± 0.01	0.22 ± 0.01	0.18 ± 0.02	0.54 ± 0.01	0.5 ± 0.04	**0.17 ± 0.01**	**0.11 ± 0.02**	0.16 ± 0.02	0.19 ± 0.03	0.15 ± 0.01	0.16 ± 0.01
*C22:1ω9*	0.21 ± 0.04	0.13 ± 0.01	**0.19 ± 0.03**	**0.08 ± 0.01**	0.41 ± 0.03	0.34 ± 0.01	0.08 ± 0.01	0.07 ± 0.02	0.08 ± 0.01	0.12 ± 0.03	0.06 ± 0.01	0.1 ± 0.02
*C22:4ω6*	**0.99 ± 0.11**	**0.31 ± 0.01**	**2.08 ± 0.08**	**0.5 ± 0.01**	**2.22 ± 0.17**	**0.75 ± 0.04**	**1.81 ± 0.1**	**0.49 ± 0.02**	**2.14 ± 0.11**	**0.59 ± 0.06**	**1.84 ± 0.1**	**0.52 ± 0.03**
*C22:5ω6*	**0.04 ± 0.02**	**0**	**0.13 ± 0.01**	**0.02 ± 0.01**	**0.05 ± 0.01**	**0**	**0.12 ± 0.01**	**0.04 ± 0.01**	**0.16 ± 0.02**	**0.05 ± 0.00**	**0.15 ± 0.02**	**0.04 ± 0.01**
*C22:5ω3*	**0.14 ± 0.04**	**0.33 ± 0.04**	**0.15 ± 0.01**	**0.43 ± 0.02**	**0.21 ± 0.01**	**0.84 ± 0.23**	**0.19 ± 0.02**	**0.44 ± 0.03**	**0.2 ± 0.02**	**0.53 ± 0.05**	**0.23 ± 0.05**	**0.47 ± 0.03**
*C22:6ω3*	**13.84 ± 0.31**	**15.52 ± 0.4**	**12.74 ± 0.36**	**16.44 ± 0.32**	9.43 ± 0.27	10.38 ± 0.62	**14.4 ± 0.37**	**19 ± 0.57**	**12.6 ± 0.4**	**16.35 ± 0.83**	**15.07 ± 0.71**	**18.07 ± 0.28**
*C24:0*	0.15 ± 0.03	0.19 ± 0.02	**0.07 ± 0.01**	**0.02 ± 0.02**	0.41 ± 0.02	0.31 ± 0.13	**0.08 ± 0.01**	**0**	**0.05 ± 0.01**	**0**	0.03 ± 0.01	0


*Values in bold reflect differences that reached statistical significance (*P* < 0.05) comparing both experimental groups with Student’s *t*-test.*

## Discussion

Since the introduction of FOLE for the treatment of IFALD, some have questioned whether FOLE alone is sufficient to support growth and neurocognitive development in infants and children. These concerns arise primarily from the long-held belief that the O3FA ALA and the O6FA LA are required to sustain human growth and development (Das, 2006). Previous studies have shown that DHA and ARA, the downstream metabolites of ALA and LA, are sufficient to support growth and reproduction in mice (Strijbosch et al., 2008; Le et al., 2009; Nehra et al., 2012; Harauma et al., 2017). To our knowledge, this is the first study demonstrating that mice treated over 10 generations with a 20:1 ratio of DHA/ARA as the sole source of polyunsaturated fatty acids demonstrate equivalent visual, motor, cognitive, and social performance when compared to chow-fed controls. This work further supports the recent findings of Harauma et al. (2017), who proved that DHA and ARA are sufficient to allow the proper development of brain structure and function in delta-6-desaturase knockout mice. Altogether, these findings question the notion of the true essentiality of ALA and LA for cognitive development and growth.

Docosahexaenoic acid comprises 10–20% of the total fatty acid composition in the brain, whereas 9% is present in the form of ARA (McNamara and Carlson, 2006). The levels of DHA in the brain increase during development and play a critical role during the growth spurt that occurs in the last trimester of pregnancy and the early postnatal period up to 2 years of age (Svennerholm, 1968; Martinez, 1992). DHA is a major component of the phospholipid in the outer membrane of photoreceptors and has significant effects on neurotransmitters involved in the signal transduction process, rhodopsin activation, rod and cone development, neuronal dendritic connectivity, and functional maturation of the central nervous system (Rotstein et al., 1997). DHA and ARA serve as bioactive molecules that regulate gene expression by acting on nuclear receptors or transcription factors, and by activating pathways of signal transduction (Jazurek et al., 2008). EFA also play roles in neurotransmitter content and its corresponding electrophysiological correlates, protection from oxidative stress, and modulation of gene expression of the developing retina and brain (Innis, 2007). Findings from the present study suggest a significant change in the percentage fatty acid distribution of the brain. Although DHA levels remained within those described in the literature, those seen in mice fed the DHA/ARA diet were significantly higher than those seen in chow-fed mice (McNamara and Carlson, 2006). Levels of EPA also showed a significant increase in DHA/ARA-fed mice, likely the result of retroconversion from its downstream metabolite, DHA (Brossard et al., 1996; Anez-Bustillos et al., 2017).

Janssen et al. (2015) compared the neurological function of mice fed an O3FA-adequate diet (3.83% of total fatty acids from a mixture of ALA and DHA) to those fed an O3FA-deficient diet (0.05% of total fatty acids from ALA and DHA). This study found that pubertal mice fed the O3FA-adequate diet demonstrated superior motor coordination compared to the O3FA-deficient group. In adulthood, mice treated with the O3FA-adequate diet showed increased sensorimotor integration and spatial memory as well as increased exploratory behavior when compared to the O3FA-deficient group. Notably the O3FA-adequate diet utilized by Janssen et al. (2015) contained significant amounts of ALA and LA (2.55% and 15.7% of total fat calories, respectively), whereas our dietary treatment consisted of primarily DHA and ARA (20:1) and no ALA and LA. A more strict approach is that used by Harauma et al. (2017) in which delta-6-desaturase knockout mice were artificially fed diets that differed in their amount of DHA, ARA, and EPA or their combinations, to better investigate their effects while being unable to metabolize ALA and LA. Under these conditions, it was found that only DHA and ARA were both required for normal brain development, whereas EPA, either alone or in combination with ARA, was not.

Mice fed the DHA/ARA diet had significantly shorter latency times per trial in the rotarod test for ataxia and balance, despite having grip strengths that were similar between both experimental groups (data not shown). However, the fact that animals from both groups performed better in each successive trial compared to the preceding one supports the conservation of their motor coordination learning capacity. A possible limitation to these testing conditions is the use of the same sized rod and speed to test mice with dissimilar scores at baseline. Lalonde et al. (1995) proposed a solution to this problem by manipulating the rods’ size and allowing it to rotate at two different speeds. We did attempt to adjust performance based on baseline characteristics by means of statistical tests, but still observed improved sensorimotor learning in the chow-fed animals. Our results differ from those of others that have assessed the effects of O3FA supplementation in motor skills in rodents. Coluccia et al. (2009) found improved rotarod performances in rats supplemented with O3FA during their development compared with control animals, although no effect was noticed under accelerating conditions. However, results from the mentioned study come from tests performed at a relatively younger age compared to that of our experiments. Similarly, acyl-CoA synthetase 6-deficient mice, which have significantly lower levels of DHA and higher levels of ARA in the brain, showed a trend toward reduced rotarod performance at 12 months of age (Fernandez et al., 2018). These findings also are in conflict with those seen in our study, although mice tested were significantly older. Future work should directly address the possible neuroprotective effects of DHA-enriched diets on the aging brain and its impact on function.

Additionally, we found that mice treated with the DHA/ARA diet demonstrated less neophobia (increased time on the open arms of the plus maze) when compared to chow-fed controls, a finding that has been previously suggested in rat models (Pérez et al., 2013; Gonzales et al., 2015; Pusceddu et al., 2015). Pusceddu et al. (2015) demonstrated that female rats supplemented with a mixture of DHA and EPA at 2–12 days of life demonstrated a dose-dependent reduction in anxiety as measured by the elevated plus maze. In a study by Pérez et al. (2013), adult male rats were treated with supplemental DHA and EPA versus saline control over a period of 3 weeks and subjected to a restraint-stress protocol. Animals treated with the O3FA mixture were found to have decreased response to stress as measured by open field and elevated plus-maze tests. In humans, O3FA have been associated with some benefits in the treatment of anxiety or anxiety-related disorders (Barbadoro et al., 2013; Haberka et al., 2013; Vesco et al., 2015). In a randomized controlled trial, healthy young adult students treated with 12 weeks of O3FA supplementation (2085 mg EPA and 348 mg DHA daily) experienced lower Beck Anxiety Inventory scores when compared to control students who received placebo (Kiecolt-Glaser et al., 2011).

Although tests assessing spatial learning and memory showed no differences between both experimental groups, working short-term memory evaluated by the NOR test showed a trend toward improved results in mice from the CHOW group. These results are conflicting with those seen in the literature, although available data comes mostly from diseased or transgenic models. In animals, there is data supporting O3FA supplementation to prevent the cognitive decline associated with several diseases. Hooijmans et al. (2012) performed a systematic review and meta-analysis that found a beneficial effect of O3FA on cognition in animal models of Alzheimer’s disease. A strength of this review stems from the inclusion of studies that provided O3FA for prolonged periods of time. This is particularly important as the lack of positive results, particularly in human studies, has been attributed to short exposure periods. A neuroprotective cognitive effect has also been shown in rats subjected to traumatic brain injury (Mills et al., 2011). Conversely, data on the neurocognitive effects of DHA and EPA supplementation from human studies are mixed. A review by Cederholm et al. (2013) concluded that in healthy individuals, even though data from epidemiologic studies suggest a beneficial effect, that from interventional trials is null. We hypothesize that the results obtained in the present study, having been obtained from healthy mice, correlate with those seen in healthy humans.

The metabolism of an enteral DHA-based diet is not the same as that of a parenteral FOLE, and this is recognized as a limitation of this study. As we do detect changes in the distribution of fatty acids in different regions of the brain, future work should analyze the impact this has on the development and plasticity of brain circuits. The present manuscript lacks data on whether the brain itself developed normally. Additionally, we acknowledge that the comparison of neurocognitive outcomes in the short life cycle of mice may not be directly translatable to those of infants and children long term. The fact that chow-fed mice were not inbred for multiple generations poses an additional limitation. We anticipate that results would not have changed significantly as animals from this group were obtained from facilities in which the diet provided is similar to the chow formula used in our experiments. Feeding mice for multiple generations with a diet containing DHA and ARA as the sole source of polyunsaturated fatty acids represents an extreme model that proves that these LC-PUFA are sufficient to support gross visual, cognitive, motor, and social development in mice. Additionally, having studied the effect of this diet after multiple generations suggests a lack of negative epigenetic effects. These results lend support to the use of fish oil-based parenteral nutrition formulations with regard to neurocognitive development in infants and children. Future work may further elucidate the neurodevelopmental outcomes of infants and children supported by long-term FOLE.

## Author Contributions

SC, AO, MF, KG, and MP contributed to the study conception and design. SC, AO, NA, GG, MB, LA-B, MC, EC, and PN conducted the study and did the data acquisition. PM did the data analysis. SC, MB, LA-B, MF, and MP did the drafting of the manuscript. SC, AO, MB, LA-B, MF, and MP interpreted the data. All authors reviewed the article and were involved in the final version of the manuscript.

## Conflict of Interest Statement

A license agreement for the use of Omegaven has been signed by Boston Children’s Hospital and Fresenius Kabi. MP and KG hold an issued patent on the treatment of parenteral nutrition-associated liver disease. They both serve on the Scientific Advisory Boards for Pronova-BASF. KG also serves on the Pharmaceutical Advisory Board for B. Braun United States. The remaining authors declare that the research was conducted in the absence of any commercial or financial relationships that could be construed as a potential conflict of interest.
